# Draft Genome Sequence of the Acidophilic, Halotolerant, and Iron/Sulfur-Oxidizing *Acidihalobacter prosperus* DSM 14174 (Strain V6)

**DOI:** 10.1128/genomeA.01469-16

**Published:** 2017-01-19

**Authors:** Himel Nahreen Khaleque, Joshua P. Ramsay, Riley J. T. Murphy, Anna H. Kaksonen, Naomi J. Boxall, Elizabeth L. J. Watkin

**Affiliations:** a CHIRI Biosciences, School of Biomedical Sciences, Curtin University, Perth, Australia; b CSIRO Land and Water, Perth, Australia

## Abstract

The principal genomic features of *Acidihalobacter prosperus* DSM 14174 (strain V6) are presented here. This is a mesophilic, halotolerant, and iron/sulfur-oxidizing acidophile that was isolated from seawater at Vulcano, Italy. It has potential for use in biomining applications in regions where high salinity exists in the source water and ores.

## GENOME ANNOUNCEMENT

*Acidihalobacter prosperus* (previously known as *Thiobacillus prosperus*) is a Gram-negative, halotolerant, acidophilic, mesophilic, and chemolitho-autotrophic bacterium capable of oxidizing both iron and reduced sulfur compounds (1). The type strain, *A. prosperus* DSM 5130, was isolated from a marine geothermal field in Italy. It requires a minimum of 0.04 M Cl^-^ for growth and tolerates up to 0.6 M Cl^-^ (1). *A. prosperus* DSM 14174 was isolated from a shallow acidic pool by the shore of Baia de Levant, Aelion Islands of Vulcano, Italy (2). Like *A. prosperus* DSM 5130, it does not grow in the absence of salt (3). It has been used in salt-rich systems for the active biomining of metal sulfide ores (4).

Total DNA extracted from *A. prosperus* DSM 14174 was sequenced using Illumina MiSeq (204,485 paired-end 300 bp × 2 reads) and PacBio RS single-molecule real-time (SMRT) sequencing technologies (96,369 reads postfilter, 11,756 bp mean length). *De novo* hybrid assembly using SPAdes 3.9.0 (5) produced a circular 162,484-bp plasmid (~32-fold coverage) and two chromosome fragments of 2,607,071 bp and 752,753 bp (~18-fold coverage). The two chromosome fragments were scaffolded using SSPACE-LongRead version 1.1 (6), producing a circular chromosome of 3,363,634 bp (with one gap). The genome has a G+C content of 62.2%. The NCBI Prokaryotic Genome Annotation Pipeline version 3.3 and GeneMarkS+ were used for annotation. The genome contains 46 tRNA sequences, one rRNA operon, and 3,194 protein-coding genes.

Genome analysis of *A. prosperus* DSM 14174 confirmed the presence of the previously reported *rus* operon known to be involved in iron oxidation (3). Also present were genes coding for subunits SoxAX, SoxB, and SoxYZ of the sulfur oxidation system (7), as well as those for sulfur metabolism through hydrogen sulfide biosynthesis (8). Furthermore, there were genes encoding proteins involved in various catalytic reactions for oxidation/reduction of sulfur as well as the transport of sulfate/sulfonate (8). Similar to the genome of the type strain* A. prosperus* DSM 5130, the genome of *A. prosperus* DSM 14174 contains a complete set of genes for carbon dioxide fixation via the Calvin-Benson-Bassham cycle, as well as those for the Nif complex for nitrogen fixation, chemotaxis, and formation of a polar flagellum (9).

The synthesis of compatible solutes, such as ectoine, sucrose, and glycine betaine, assists in the survival of bacteria under high osmotic stress (10). The genome of *A. prosperus* DSM 14174 contains genes that encode diaminobutyrate aminotransferases, diaminobutyrate acetyltransferase, ectoine synthase, and sucrose synthase. These have potential roles in ectoine and sucrose biosynthesis pathways. Genes for ABC transporters for ectoine and glycine betaine uptake were also detected.

*A. prosperus* DSM 14174 contains a single plasmid, pABPV6, which is unique to this strain. The plasmid pABPV6 contains an array of genes coding for replication and transfer proteins, transposases, DNA methyltransferases, recombinases, hydrolases, and DNA binding proteins.

### Accession number(s).

The whole-genome sequence has been deposited at DDBJ/EMBL/GenBank under the GenBank accession no. CP017448. The plasmid pABPV6 has been deposited under the GenBank accession no. CP017449. The versions described in this paper are CP017448.1 and CP017449.1, respectively.
